# On Some Physiological Researches, Respecting the Influence of the Brain on the Action of the Heart, and on the Generation of Animal Heat

**Published:** 1811-07

**Authors:** B. C. Brodie


### *On some Physiological Researches, respecting the Influence of the Brain on the Action of the Heart, and on the Generation of Animal Heat.* By Mr. B. C. Brodie, F.R.S.

(Phil. Trans.)

*Read before the Royal Society, Dec.* 20, 1810.

HAVING had the honour of being appointed, by the President of the Royal Society, to give the Croonian Lecture, I trust that the following facts and observations will be considered as tending sufficiently to promote the objects, for which the Lecture was instituted. They appear to throw some light on the mode in which the influence of the brain is necessary to the continuance of the action of the heart; and on the effect which the changes produced on the blood in respiration have on the heat of the animal body.

In making experiments on animals to ascertain how far the influence of the brain is necessary to the action of the heart, I found, that when an animal was pithed by dividing the spinal marrow in the upper part of the neck, respiration was immediately destroyed, but the heart still continued to contract circulating dark-coloured blood, and, that in some instances, from ten to fifteen minutes elapsed before its action had had entirely ceased. I further found that when the head was removed, the divided blood vessels being secured by a ligature, the circulation still continued, apparently unaffected by the entire separation of the brain. These experiments confirmed the observations of Mr. Cruikshank* and M. Bichat,† that the brain is not directly necessary to the action of the heart, and that when the functions of the brain are destroyed, the circulation ceases only in consequence of the suspension of respiration. This led me to conclude, that, if respiration was produced artificially, the heart would continue to contract for a still longer period of time after the removal of the brain. The truth of this conclusion was ascertained by the following experiment.

*Experiment* 1.

I divided the spinal marrow of a rabbit in the space between the occiput and atlas, and having made an opening into the trachea, fitted into it a tube of elastic gum, to which was connected a small pair of bellows, so constructed that the lungs might be inflated, and then allowed to empty themselves. By repeating this process once in five seconds, the lungs being each time fully inflated with fresh atmospheric air, an artificial respiration was kept up. I then secured the blood-vessels in the neck, and removed the head by cutting through the soft part above the ligature, and separating the occiput from the atlas. The heart continued to contract, apparently with as much strength and frequency as in a living animal. I examined the blood in the different sets of vessels, and found it dark-coloured in the venæ cavæ and pulmonary artery, and of the usual florid red colour in the pulmonary veins and aorta. At the end of twenty-five minutes from the time of the spinal marrow being divided, the action of the heart became fainter, and the experiment was put an end to.

With a view to promote the inquiry instituted by the Society for promoting the knowledge of animal chemistry respecting the influence of the nerves on the secretions‡, I endeavoured to ascertain whether they continued after the influence of the brain was removed. In the commencement of the experiment I emptied the bladder of its contents by pressure; at the end of the experiment the bladder continued empty.

This experiment led me to conclude, that the action of the heart might be made to continue after the brain was removed,

* Philosophical Transactions 1795.

† Récherches Physiologiques sur la Vie et la Mort.

‡ Philosophical Transactions for 1809.

by means of artificial respiration, but that under these circumstances the secretion of urine did not take place. It appeared, however, desirable to repeat the experiment on a larger and less delicate animal, and that, in doing so, it would be right to ascertain, whether under these circumstances, the animal heat was kept up to the natural standard.

*Experiment* 2.

I repeated the experiment on a middle sized dog. The temperature of the room was 63° of Fahrenheit's thermometer. By having previously secured the carotid and vertebral arteries, I was enabled to remove the head with little or no hæmorrhage. The artificial respirations were made about twenty-four times in a minute. The heart acted with regularity and strength.

At the end of 30 minutes from the time of the spinal marrow being divided, the heart was felt through the ribs contracting 76 times in a minute.

At 35 minutes the pulse had risen to 84 in a minute.

At one hour and 30 minutes the pulse had risen to 88 in a minute.

At the end of two hours it had fallen to 70, and at the end of two hours and a half to 35 in a minute, and the artificial respiration was no longer continued.

By means of a small thermometer with an exposed bulb, I measured the animal heat at different periods.

At the end of an hour the thermometer in the rectum had fallen from 100° to 94°.

At the end of two hours, a small opening being made in the parietes of the thorax, and the ball of the thermometer placed in contact with the heart, the mercury fell to 86°, and half an hour afterwards in the same situation it fell to 78°.

In the beginning of the experiment I made an opening into the abdomen, and having passed a ligature round each ureter about two inches below the kidney, brought the edges of the wound in the abdomen together by means of sutures. At the end of the experiment no urine was collected in the ureters above the ligatures.

On examining the blood in the different vessels, it was found of a florid red colour in the arteries, and of a dark colour in the veins, as under ordinary circumstances.

During the first hour and a half of the experiment, there were constant and powerful contractions of the muscles of the trunk and extremities, so that the body of the animal was moved moved in a very remarkable manner, on the table on which it lay, and twice there was a copious evacuation of fæces.

*Experiment 3.*

The experiment was repeated on a rabbit. The temperature of the room was 60°. The respirations were made from 30 to 35 in a minute. The actions of the heart at first were strong and frequent: but at the end of one hour and forty minutes the pulse had fallen to 24 in a minute.

The blood in the arteries was seen of a florid red, and that in the veins of a dark colour.

A small opening was made in the abdominal muscles, through which the thermometer was introduced into the abdomen, and allowed to remain among the viscera.

At the end of an hour the heat in the abdomen had fallen from 100° to 89°. At the end of an hour and forty minutes in the same situation the heat had fallen to 85°, and when the bulb of the thermometer was placed in the thorax in contact with the lungs, the mercury fell to 82°.

It has been a very generally received opinion, that the heat of warm-blooded animals is dependent on the chemical changes produced on the blood by the air in respiration. In the two last experiments, the animals cooled very rapidly, notwithstanding the blood appeared to undergo the usual changes in the lungs, and I was therefore induced to doubt whether the above mentioned opinion respecting the source of animal heat is correct. No positive conclusions, however, could be deduced from these experiments. If animal heat depends on the changes produced on the blood by the air in respiration, its being kept up to the natural standard, or otherwise, must depend on the quantity of air inspired, and on the quantity of blood passing through the lungs in a given space of time: in other words, it must be in proportion to the fullness and frequency of the pulse, and the fullness and frequency of the inspirations. It therefore became necessary to pay particular attention to these circumstances.

*Experiment 4.*

The experiment was repeated on a dog of a small size, whose pulse was from 130 to 140 in a minute, and whose respirations, as far as I could judge, were performed from 30 to 35 times in a minute.

The temperature of the room was 63°. The heat in the rectum of the animal at the commencement of the experiment was 99°. The artificial inspirations were made to correspond as as nearly as posssible to the natural inspirations both in fullness and frequency.

At 20 minutes from the time of the dog being pithed, the heart acted 140 times in a minute with as much strength and regularity as before: the heat in the rectum had fallen to 96½.

At 40 minutes the pulse was still 140 in a minute: the heat in the rectum 92½.

At 55 minutes the pulse was 112, and the heat in the rectum 90°.

At one hour and 10 minutes the pulse beat 90 in a minute, and the heat in the rectum was 88°.

At one hour and 25 minutes the pulse had sunk to 30, and the heat in the rectum was 85°. The bulb of the thermometer being placed in the bag of the pericardium, the mercury stood at 85°, but among the viscera of the abdomem it rose to 87½.

During the experiment there were frequent and violent contractions of the voluntary muscles, and an hour after the experiment was begun, there was an evacuation of fæces.

### *Experiment* 5.

The experiment was repeated on a rabbit, whose respirations, as far as I could judge, were from 30 to 40 in a minute, and whose pulse varied from 130 to 140 in a minute. The temperature of the room was 57°. The heat in the rectum, at the commencement of the experiment, was 101½. The artificial respirations were made to resemble the natural respirations as much as possible, both in fullness and frequency.

At 15 minutes from the time of the spinal marrow being divided, the heat in the rectum had fallen to 98½.

At the end of half an hour the heart was felt through the ribs, acting strongly 140 times in a minute.

At 45 minutes the pulse was still 140; the heat in the rectum was 94.

At the end of an hour the pulse continued 140 in a minute; the heat in the rectum was 92°; among the viscera of the abdomen 94°; in the thorax, between the lungs and pericardium, 92°.

During the experiment, the blood in the femoral artery was seen to be of a bright florid colour, and that in the femoral vein of a dark colour, as usual.

The rabbit voided urine at the commencement of the experiment; at the end of the experiment, no urine was found in the bladder.

### *Experiment* 6.

I procured two rabbits of the same colour, but one of them was was about one-fifth smaller than the other. I divided the spinal marrow of the larger rabbit between the occiput and atlas. Having secured the vessels in the neck, and removed the head, I kept up the circulation by means of artificial respiration as in the former experiments. The respirations were made as nearly as possible similar to natural respirations.

In 23 minutes after the spinal marrow was divided, the pulse was strong, and 130 in a minute: the ball of the thermometer being placed among the viscera of the abdomen, the mercury stood at 96°.

At 34 minutes the pulse was 120 in a minute; the heat in the abdomen was 95°.

At the end of an hour the pulse could not be felt, but on opening the thorax the heart was found acting, but slowly and feebly. The heat in the abdomen was 91°; and between the lobes of the right lung 88°.

During the experiment, the blood in the arteries and veins was seen to have its usual colour.

In this therefore, as in the preceding experiments, the heat of the animal sunk rapidly, notwithstanding the continuance of the respiration. In order to ascertain whether any heat at all was generated by this process, I made the following comparative experiment. The temperature of the room being the same, I killed the smaller rabbit by dividing the spinal marrow between the occiput and atlas. In consequence of the difference of size, *cœteris paribus*, the heat in this rabbit ought to diminish more rapidly than in the other; and I therefore examined its temperature at the end of 52 minutes, considering that this would be at least equivalent to examining that of the larger rabbit at the end of an hour. At 52 minutes from the time of the smaller rabbit being killed, the heat among the viscera of the abdomen was 92°, and between the lobes of the right lung it was 91°. From this experiment, therefore, it appeared not only that no heat was generated in the rabbit, in which the circulation was maintained by artificial respiration, but that it even cooled more rapidly than the dead rabbit.

At the suggestion of Professor Davy, who took an interest in the inquiry, I repeated the foregoing experiment on two animals, taking pains to procure them more nearly of the same size and colour.

### *Experiment* 7.

I procured two large full grown rabbits, of the same colour, and so nearly equal in size, that no difference could be detected by the eye.

The

The temperature of the room was 57°, and the heat in the rectum of each rabbit previous to the experiment was 100½.

I divided the spinal marrow in one of them, produced artificial respiration, and removed the head after having secured the vessels in the neck. The artificial respirations were made about 35 times in a minute.

During the first hour, the heart contracted 144 times in a minute.

At the end of an hour and a quarter the pulse had fallen to 136 in a minute, and it continued the same at the end of an hour and a half. At the end of an hour and forty minutes the pulse had fallen to 90 in a minute, and the artificial respiration was not continued after this period.

Half an hour after the spinal marrow was divided, the heat in the rectum had fallen to 97°.

At 45 minutes the heat was 95½.

At the end of an hour the heat in the rectum was 94°.

At an hour and a quarter it was 92°.

At an hour and a half it was 91°.

At an hour and forty minutes, the heat in the rectum was 90½, and in the thorax, within the bag of the pericardium, the heat was 87½.

The temperature of the room being the same, the second rabbit was killed by dividing the spinal marrow, and the temperature was examined at corresponding periods.

Half on hour after the rabbit was killed, the heat in the rectum was 99°.

At 45 minutes it had fallen to 98°.

At the end of an hour the heat in the rectum was 96½.

At an hour and a quarter it was 95°.

At an hour and a half it was 94°.

At an hour and forty minutes the heat in the rectum was 93°, and in the bag of the pericardium 90½.

The following table will shew the comparative temperature of the two animals at corresponding periods.

| Time. | Rabbit with artificial respiration. | | Dead Rabbit. | |
|---|---|---|---|---|
| | Therm. in the Rectum. | Therm. in the Pericardium. | Thermom. in the Rectum. | Therm. in the Pericardium. |
| Before the Experiment | 100½ | | 100½ | |
| 30 min. | 97 | | 99 | |
| 45 — | 95½ | | 98 | |
| 60 — | 94 | | 96½ | |
| 75 — | 92 | | 95 | |
| 90 — | 91 | | 94 | |
| 100 — | 90½ | 87½ | 93 | 90½ |

In this experiment, the thorax, even in the dead animal, cooled more rapidly than the abdomen. This is to be explained by the difference in the bulk of these two parts. The rabbit in which the circulation was maintained by artificial respiration, cooled more rapidly than the dead rabbit, but the difference was more perceptible in the thorax than in the rectum. This is what might be expected, if the production of animal heat does not depend on respiration, since the cold air by which the lungs were inflated, must necessarily have abstracted a certain quantity of heat, particularly as its influence was communicated to all parts of the body, in consequence of the continuance of respiration.

It was suggested that some animal heat might have been generated, though so small in quantity as not to counterbalance the cooling powers of the air thrown into the lungs. It is difficult or impossible, to ascertain with perfect accuracy, what effect cold air thrown into the lungs would have on the temperature of an animal under the circumstances of the last experiment, independently of any chemical action on the blood: since, if no chemical changes were produced, the circulation could not be maintained, and if the circulation ceased, the cooling properties of the air must be more confined to the thorax, and not communicated in an equal degree to the more distant parts. The following experiment, however, was instituted, as likely to afford a nearer approximation to the truth, than any other that could be devised.

*Experiment* 8.

I procured two rabbits of the same size and colour: the temperature of the room was 64°. I killed one of them by dividing the spinal marrow, and immediately, having made an opening into the left side of the thorax, I tied a ligature round the base of the heart, so as to stop the circulation. The wound in the skin was closed by a suture. An opening was then made into the trachea, and the apparatus for artificial respiration being fitted into it, the lungs were inflated, and then allowed to collapse as in the former experiment, about 36 times in a minute. This was continued for an hour and a half, and the temperature was examined at different periods. The temperature of the room being the same, I killed the second rabbit in the same manner, and measured the temperature at corresponding periods. The comparative temperature of the two dead animals, under these circumstances, will be seen in the following table.

| Time. | Dead Rabbit whose lungs were inflated. | | Dead Rabbit whose lungs were not inflated. | |
|---|---|---|---|---|
| | Therm. in the Rectum. | Therm. in the Thorax. | Thermom. in the Rectum. | Therm. in the Thorax. |
| Before the Experiment | 100½ | | 100 | |
| 30 min. | 97 | | 98 | |
| 45 — | 95½ | | 96 | |
| 60 — | 94 | | 94½ | |
| 75 — | 92½ | | 93 | |
| 90 — | 91 | 86 | 91½ | 88½ |

In this last experiment, as may be seen from the above table, the difference in the temperature of the two rabbits, at the end of an hour and a half, in the rectum, was half a degree, and in the thorax two degrees and a half; whereas, in the preceding experiment, at the end of an hour and forty minutes, the difference in the rectum was 2½ degrees, and in the thorax 3 degrees. It appears, therefore, that the rabbit in which the circulation was maintained by artificial respiration, cooled more rapidly on the whole than the rabbit whose lungs were inflated in the same manner after the circulation had ceased. This is what might be expected, if no heat was produced by the chemical action of the air on the blood; since in the last case the cold air was always applied to the same surface, but in the former it was applied always to fresh portions of blood, by which its cooling powers were communicated to the more distant parts of the body.

In the course of the experiments which I have related, I was much indebted to several members of the Society for promoting the Knowledge of Animal Chemistry, for many important suggestions which have assisted me in prosecuting the inquiry. Mr. Home, at my request, was present at the seventh experiment. Dr. E. N. Bancroft was present at, and assisted me in the second experiment; and Mr. William Brande lent me his assistance in the greater part of those which were made. I have been further assisted in making the experiments by Mr. Broughton, surgeon of the Dorsetshire Regiment of Militia, and Mr. Richard Rawlins, and Mr. Robert Gatcombe, students in surgery.

I have selected the above from a great number of similar experiments, which it would be needless to detail. It is sufficient to state, that the general results were always the same; and that whether the pulse was frequent or slow, full or small, or whether the respirations were frequent or otherwise, there was no perceptible difference in the cooling of the animal.

From

From the whole we may deduce the following conclusions:

1. The influence of the brain is not directly necessary to the action of the heart.

2. When the brain is injured or removed, the action of the heart ceases, only because respiration is under its influence, and if under these circumstances respiration is artificially produced, the circulation will still continue.

3. When the influence of the brain is cut off, the secretion of urine appears to cease, and no heat is generated; notwithstanding the functions of respiration, and the circulation of the blood continue to be performed, and the usual changes in the appearance of the blood are produced in the lungs.

4. When the air respired is colder than the natural temperature of the animal, the effect of respiration is not to generate, but to diminish animal heat.

### *Addition to the Croonian Lecture for the Year* 1810.

In the experiments formerly detailed, where the circulation was maintained by means of artificial respiration after the head was removed, I observed that the blood, in its passage through the lungs, was altered from a dark to a scarlet colour, and hence I was led to conclude that the action of the air produced in it changes analogous to those, which occur under ordinary circumstances. I have lately, with the assistance of my friend Mr. W. Brande, made the following experiment, which appears to confirm the truth of this conclusion.

An elastic gum bottle, having a tube and stop-cock connected with it, was filled with about a pint of oxygen gas. The spinal marrow was divided in the neck of a young rabbit, and the blood vessels having been secured, the head was removed, and the circulation was maintained by inflating the lungs with atmospheric air for five minutes, at the end of which time the tube of the gum bottle was inserted into the trachea, and carefully secured by a ligature, so that no air might escape. By making pressure on the gum bottle, the gas was made to pass and repass into and from the lungs about thirty times in a minute. At first, the heart acted one hundred and twenty times in a minute, with regularity and strength; the thermometer, in the rectum, rose to 100°. At the end of an hour, the heart acted as frequently as before, but more feebly; the blood in the arteries was very little more florid than that in the veins; the thermometer in the rectum had fallen to 93°. The gum bottle was then removed. On causing a stream of the gas which it contained, to pass through lime-water, the presence of carbonic acid was indicated cated by the liquid being instantly rendered turbid. The proportion of carbonic acid was not accurately determined; but it appeared to form about one-half of the quantity of gas in the bottle.

B. C BRODIE.

